# Recurrent Osteosarcoma Presenting as Hepatic Metastasis: A Case Report

**DOI:** 10.7759/cureus.73087

**Published:** 2024-11-05

**Authors:** Abdullah Imran, Muhammad Qasim Naeem, Noor Fatima, Khadija Muneer, Kashif Siddique

**Affiliations:** 1 Radiology, Shaukat Khanum Memorial Cancer Hospital and Research Centre, Lahore, PAK; 2 Medical Unit 1, Services Hospital Lahore, Lahore, PAK

**Keywords:** extrapulmonary metastasis, hepatic metastasis, metastatic osteosarcoma, osteosarcoma, recurrent osteosarcoma

## Abstract

Osteosarcoma is a malignant tumor, derived from primitive bone-forming mesenchymal cells, and its aggressive nature often leads to significant morbidity and mortality, with amputation being routinely performed in localized cases. Post-amputation morbidity has a significant impact on the quality of life of the affected individuals. The tumor frequently metastasizes to the lungs and bones, and widespread disease remains the leading cause of most patient deaths. Other metastatic sites are extremely rare. We report a rare case of a young boy who had developed osteosarcoma and was surgically treated, without any metastatic disease. Two years later, the patient developed an abdominal mass, which was diagnosed as metastatic osteosarcoma to the liver.

## Introduction

Osteosarcoma is a malignant bone-forming tumor with a mesenchymal origin, classified into primary and secondary types. Primary osteosarcoma mainly affects adolescents and young adults and is the most common primary bone cancer in this age group, while secondary osteosarcoma is found in the elderly, usually due to malignant degeneration of Paget disease or benign bone tumors, or as a complication of radiotherapy [1-4]. Primary osteosarcoma shows a slightly increased predominance in male children and typically occurs before age 20; this coincides with pubertal growth spurt and suggests a close relationship between rapid bone proliferation and osteosarcoma [3]. The tumor most commonly arises in the metadiaphysis of long bones such as the femur (42%), tibia (19%), and humerus (10%) and can often extend to involve the epiphysis and the adjacent joint since the growth plate does not act as an effective barrier [3].

Majority of osteosarcomas are high-grade lesions showing a tendency for early metastases, with approximately 20% of patients having distant metastases at the time of presentation [4]. There is no current optimal standard of management due to variations in different metastatic patterns with significantly different outcomes. For example, lung metastases, the most frequent kind, have a better prognosis than multifocal osseous dissemination which has a very poor outcome [4]. Survival is dependent on complete resection of metastatic deposits. All of these factors compound the difficulty of surveillance of osteosarcoma. In addition, careful consideration must be given to the radiation and contrast burden on the patient since many of them are young children.

Treatment options involve neoadjuvant chemotherapy and surgery. This is usually a wide local excision to reduce the risk of tumor recurrence. However, depending on the response to neoadjuvant treatment, limb-sparing or joint-sparing surgeries may be considered to preserve function. Surgery is followed by postoperative adjuvant chemotherapy with common chemotherapeutic agents including doxorubicin, cisplatin, methotrexate, ifosfamide, and etoposide. Radiotherapy is considered in cases with relapsed or refractory disease [1].

Disease prognosis depends on several factors such as initial tumor size, presence of metastatic disease, and response to neoadjuvant chemotherapy [4]. Survival rates for nonmetastatic and metastatic disease are 60%-70% and 10%-30%, respectively [2]. About 40% of the patients with nonmetastatic disease, at the time of presentation, eventually go on to develop metastases at later stages [1].

The most common sites of metastatic spread are the lungs, followed by the skeletal system [1,4,5-7]. Data on hepatic metastases is rather scarce with only a handful of cases found in the literature [8-12]. We report a case of recurrent metastatic osteosarcoma in the liver, in a teenage boy, who was initially surgically treated for osteosarcoma of the humerus.

## Case presentation

The patient was a 14-year-old male, a grade-seven student, who presented to our outpatient department with a five-month history of painful swelling in the right shoulder. He reported that the pain was severe in intensity and worsened with movement. The pain was also more pronounced at night.

On examination, there was a 120 mm mass over the right proximal humerus, with slightly prominent forearm veins, but no neurovascular abnormalities.

**Figure 1:**
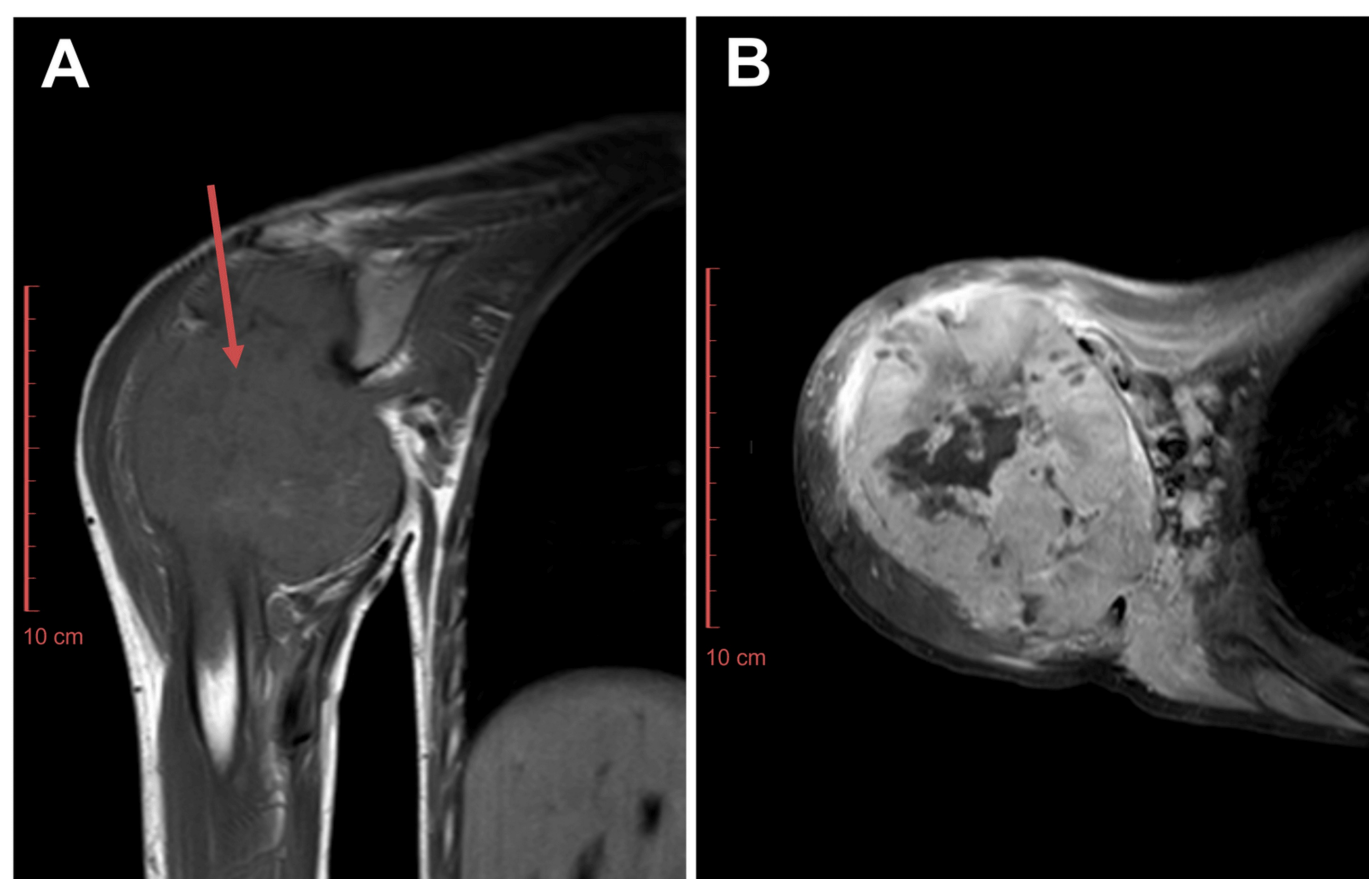
Baseline MRI right shoulder joint, (A) T1 coronal and (B) T1 postcontrast axial images showing a large mass (arrow in A) arising from the proximal metadiaphysis of the right humerus. Histopathology revealed osteosarcoma

**Figure 2:**
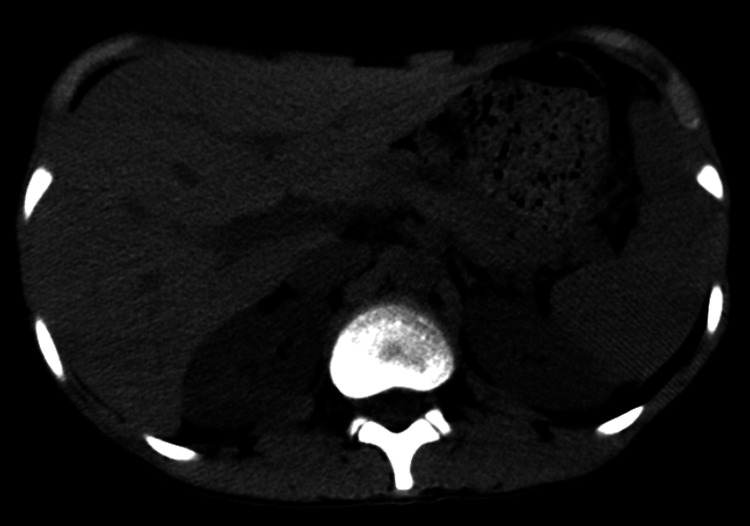
Baseline noncontrast CT chest axial images. Partially covered liver shows no lesions. It should be noted that a noncontrast CT is suboptimal for detecting hepatic metastases

Baseline MRI showed a large destructive bony lesion with a soft tissue component measuring 112 x 76 x 114 mm (Figure 1). The mass involved the glenohumeral joint and the medullary bone of the right humerus. Baseline CT of the thorax showed no evidence of pulmonary metastases and no disease in the partially visualized abdomen (Figure 2). Biopsy findings were consistent with osteosarcoma.

**Figure 3:**
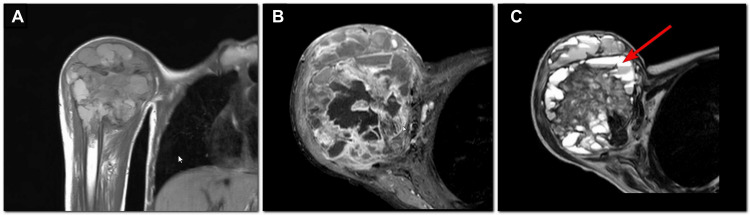
MRI right shoulder joint, (A) T1 coronal, (B) T1 postcontrast axial, and (C) T2 axial images. Follow-up scan after two cycles of chemotherapy. The mass shows posttreatment necrosis, seen as hyperintense areas with fluid-fluid levels on T2 images (arrow in C)

After a multidisciplinary team meeting, it was decided to start the patient on a chemotherapy regimen consisting of methotrexate, doxorubicin, and cisplatin. After completing two cycles of chemotherapy, a follow-up MRI showed a treatment response with necrotic changes in the tumor (Figure 3). The patient was then scheduled for a right upper limb interthoracoscapular amputation (forequarter amputation). The surgery was completed without complications, and the patient was discharged on the second postoperative day with a modified Aldrete score of 10. The patient underwent four additional cycles of chemotherapy over a six-month period, after which the patient’s status was labeled as end of treatment (EOT).

**Figure 4:**
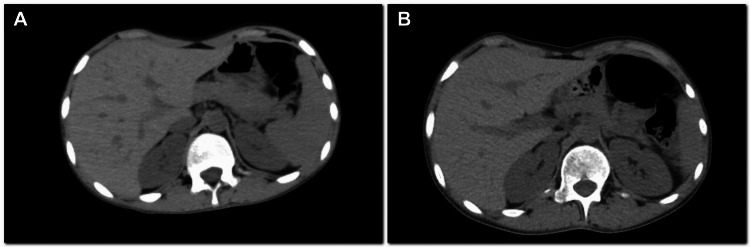
Noncontrast CT chest axial images. (A) Acquired one week after previous MRI and (B) acquired at the time of end of treatment, six months later. Partially covered liver shows no lesions

**Figure 5:**
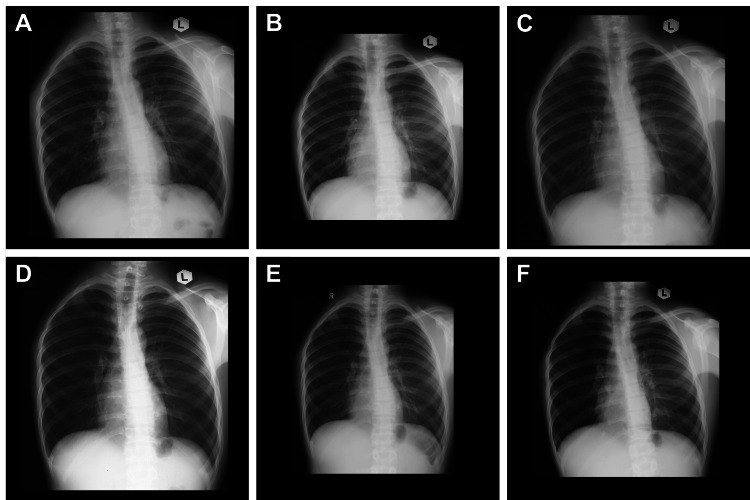
Follow-up chest X-rays for surveillance, taken at (A) one month, (B) four months, (C) seven months, (D) 10 months, (E) 12 months, and (F) 17 months after end of treatment. No evidence of pulmonary metastases

The EOT CT of the thorax showed no evidence of pulmonary metastases and no abnormalities in the partially visualized abdomen (Figure 4). The patient continued follow-up with the primary team, undergoing serial chest X-rays (Figure 5). There was no suspicion of disease progression, and no abdominal imaging was performed during this period.

**Figure 6:**
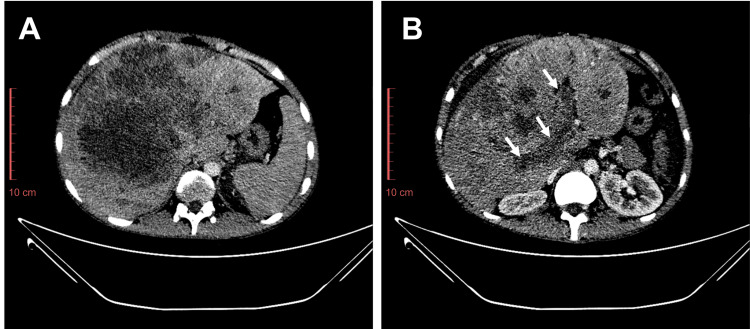
Contrast CT axial images, taken 19 months after end of treatment. (A) Enlarged liver with poorly defined hypodense mass. (B) Tumor thrombus was seen in the portal vein (arrows). Histopathology revealed metastatic osteosarcoma. No pulmonary metastases were seen (not shown)

Nineteen months after the EOT, the patient presented with a history of fever and epigastric pain for 5-6 days. CT scan revealed an enlarged liver with a poorly defined, hypodense mass in the right lobe measuring 162 x 147 mm, multiple additional smaller hypodense lesions in both lobes, and moderate abdominopelvic ascites (Figure 6). Histopathology of the hepatic lesion turned out to be metastatic osteosarcoma. The patient passed away three months after the diagnosis of hepatic metastasis. 

## Discussion

Bone sarcomas are a category of mesenchymal tumors characterized by considerable diversity. The major types of bone sarcomas are osteosarcoma, Ewing sarcoma, and chondrosarcoma, with each type further divided into several histological subcategories. Osteosarcoma is marked by the formation of osteoid or immature bone tissue [13] and typically develops in the metaphyseal regions of long bones; particularly in areas experiencing rapid growth [14]. Primary extraskeletal sites have also been mentioned in the literature but are rare [15-19]. Despite advancements in treatment strategies through a multidisciplinary approach, the prognosis for osteosarcoma remains poor due to its high propensity for metastases, particularly to the lungs, and its resistance to standard therapies [1,4,5]. Advances in molecular biology and genomics are shedding light on the intricate mechanisms underlying osteosarcoma, providing insights into potential targeted therapies and novel treatment approaches [1,5,13,14].

Radiological imaging plays a crucial role in the diagnosis, staging, and surveillance of osteosarcoma. Conventional X-rays and advanced imaging modalities, such as CT and MRI, are instrumental in assessing tumor extent and detecting metastases. Positron emission tomography (PET) with fluorodeoxyglucose (FDG) is particularly useful for evaluating metabolic activity and identifying micrometastases [6,7]. In the modern era, the widespread availability of diagnostic modalities and advancements in genetics have made it easier to detect various presentations of metastatic spread.

The treatment of osteosarcoma requires a multidisciplinary approach, aggressive chemotherapy, and wide local resection. Metastatic or locally recurrent osteosarcoma is an exceptionally formidable challenge that has not been fully overcome yet. Patients with these conditions often experience notably poor survival rates.

There is a high chance of local or distant recurrence after primary resection, most commonly in the lungs [4,13]. Extrapulmonary metastases, when detected, are more linked to aggressive disease progression. While recurrence of osteosarcoma as hepatic metastasis is documented, it is usually preceded by local recurrence at the primary site [8,11]. In our patient, however, hepatic metastasis developed without any prior local recurrence at the original tumor site. It should be noted that he continued to visit our hospital for regular follow-ups, where his chest radiographs consistently showed no abnormalities. However, two years after the amputation, he began experiencing abdominal pain. A CT scan was performed to investigate the cause, revealing multiple hepatic deposits, with histopathological analysis confirming their metastatic origin. The patient had previously undergone neoadjuvant chemotherapy, followed by an interthoracoscapular amputation, and received postoperative chemotherapy. He was referred to the palliative medicine department. The patient died three months after diagnosis of hepatic metastases.

## Conclusions

Recurrent osteosarcoma continues to pose significant challenges. While pulmonary metastases are the most common, it is important to assess extrapulmonary sites, such as the abdomen, potentially through routine ultrasound examinations. Clinicians should also be mindful of the rare presentations of recurrent osteosarcoma as these sites can easily be missed if only relying on routine chest X-rays for surveillance. Metastatic disease has a significant role in determining the prognosis, and up to 40% of patients without metastases at presentation eventually develop metastases later, highlighting the importance of early detection of disease spread. Looking ahead, we are hopeful that future advancements in medical research and technology will result in an effective cure for this condition. 
